# Attitudes toward and patterns of medication use among people with serious mental illness: There’s more than meets the eye

**DOI:** 10.3389/fpsyt.2023.1133140

**Published:** 2023-02-17

**Authors:** Maia Asher, David Roe, Ilanit Hasson-Ohayon

**Affiliations:** ^1^Department of Psychology, Bar-Ilan University, Ramat Gan, Israel; ^2^Department of Community Mental Health, Faculty of Social Welfare and Health Sciences, University of Haifa, Haifa, Israel

**Keywords:** attitudes toward medication, adherence to psychiatric medication, serious mental illness, recovery, narrative interviews, qualitative research

## Abstract

**Background and aim:**

There are growing concerns about the long-term effects of psychiatric medication after a major psychiatric crisis. Recent evidence shows a diverse impact of long-term use on various outcome domains, which may help explain why non-adherence is so common. In the current study we explored the subjective perceptions of factors that impact both attitudes toward and patterns of use of medication among individuals with serious mental illness (SMI).

**Method:**

Sixteen individuals with an SMI and a recognized psychiatric disability who had used psychiatric medication for at least 1 year were recruited for the study *via* mental health clinics and social media. Participants were interviewed using a semi-structured interview based on the narrative approach, focusing on attitudes toward and patterns of use of psychiatric medication. All interviews were transcribed and analyzed using thematic analysis.

**Results:**

Three discrete sequential phases emerged, each characterized by different themes referring to attitudes toward medication and patterns of use: (1) “loss of self” and a high level of medication use; (2) accumulating experiences of using/reducing/stopping medication; and (3) forming more stable attitudes toward medication and developing one’s own pattern of use. The transition between phases was dynamic in nature and represents a non-linear process. Complex interactions were generated at different phases between the related themes, which shaped attitudes toward medication and patterns of use.

**Conclusions and implications:**

The current study reveals the complex ongoing process of forming attitudes toward medication and patterns of use. Recognizing and identifying them *via* a joint reflective dialog with mental health professionals can enhance alliance, shared decision-making, and person-centered recovery-oriented care.

## Introduction

In accordance with treatment guidelines, psychiatric medication is recommended as the core treatment for individuals with serious mental illness (SMI; 1, 2). Yet approximately half of those who are prescribed medication do not adhere to treatment, according to a recent systematic review (3). Non-adherence is associated with a host of negative outcomes including symptom exacerbation, more emergency room visits and psychiatric hospitalizations, and high mortality rates (4–6). A common assumption as to why people do not use medications from which they could benefit is that they lack “insight” into the severity of their condition or the benefits of medication, or that their disorder hinders their ability to weigh the pros and cons of medication use (3, 7, 8). These assumptions have contributed to the widely held view of non-adherence as a negative phenomenon, as reflected in the identification of treatment adherence by the World Health Organization (WHO) as a topic of top priority in the use of evidence-based medicine (9), consequently encouraging the development of interventions to address this issue (10–12).

Although the negative consequences of non-adherence have long been noted, a growing body of research on the long-term impact of psychiatric medication on various life domains has challenged the simplistic assumption that adherence is necessarily a desired and rational choice (13–15). As such, there has been an increased effort to support people who choose to reduce dosage or stop medication use entirely (16, 17). In addition, the emphasis on personal recovery, self-determination, and shared decision-making (18, 19), along with consumers’ first-person accounts (20) and policy developments such as forming medication-free psychiatric wards (21), have generated a shift in focus. This shift could be described as moving from a narrow dichotomous perspective to a wider perspective in which the factors that influence attitudes toward and patterns of psychiatric medication use can be better understood.

A recent systematic review of reasons for non-adherence among people with an SMI revealed that a negative attitude toward medication was the key reason for intentional non-adherence (7). According to the Health Belief Model (22), attitudes toward medication consist of different beliefs including; perceived benefits and barriers of adherence, perceived susceptibility, and perceived severity of outcome. Despite the widely used Drug Attitude Inventory which entails two main clusters – namely, one’s subjective experience of medication and one’s general beliefs and attitudes about medication (23) – most studies refer to attitudes toward medication in a dichotomous manner (i.e., positive vs. negative). In doing so, the assessment of attitudes toward medication has struggled to capture the complexity of attitudes toward medication and changes over time in the way people weight their impact on various life domains (24, 25). Regarding medication use, qualitative studies have helped us gain a better and deeper understanding of the heterogeneity of people’s experiences with psychiatric medication (26–29). These studies have emphasized how people often choose to reduce the dosage or stop medication use entirely for a variety of reasons, including the way the medication was introduced, prescribed, perceived, and the way they evaluated its impact. These studies also revealed that the dichotomous categorizing of adherence as “yes” or “no” is simplistic and ignores the wide range of ways in which people use their medication.

There are several classic explanatory models and conceptual frameworks that have aimed to explain the complexity of medication adherence in mental health (e.g., 30–32). McCan and colleagues (33) offered the Self-Efficacy Model of Medication Adherence (SEMMA) in SMI, a theory-driven model based on a systematic review that refers to adherence and non-adherence as a continuum, instead of a dichotomous construct. In the model, core factors such as self-efficacy, perceived medication efficacy, and relationships with health professionals, as well as contextual influences such as personal issues or side effects as factors which influence the way people use their medications, were identified (33). The model emphasizes the synergistic interaction between the different variables in their total influence on the person’s adherence level; however, not being longitudinal, it cannot explain the nature of this complex interaction over time in a person’s coping process with an SMI.

As evident from the increased amount of literature on the subject, attitudes toward medication and patterns of use are complex and highly influenced by the individual’s experiences, beliefs, relationships, and preferences at a given time. The purpose of the present study was to investigate subjective perceptions of factors that impact attitudes toward and patterns of medication use, and how these two interact over time.

## Method

### Participants

Research participants were 16 individuals with an average age of 38.5 (*SD* = 11.3). Inclusion criteria included (1) having a diagnosis of an SMI such as schizophrenia/schizoaffective disorder, bipolar disorder, or major depressive disorder (based on the Mini International Neuropsychiatric Interview/MINI structured interview for psychiatric diagnosis); (2) meeting the criteria for having a psychiatric disability severe enough to compromise at least 40% of one’s functional ability as determined by a medical committee including a psychiatrist and recognized by National Insurance Institute (NII) regulations, and (3) having used a psychiatric medication at some point in their life for at least one full year after a major psychiatric crisis (anti-psychotic and/or mood stabilizers for bipolar disorder or major depression; *mean* time of medication use = 14.03 years, *SD* = 8.25). Of the 16 participants, four did not take medication at all at the time of the interviews, six took medications as prescribed on a regular basis, and the remaining six reported taking lower doses than prescribed or only at times of crisis (measured by a self-report question, see Instruments). Further sociodemographic and medical background characteristics are presented in Table 1.

**Table 1:** Sociodemographic and medical background characteristics of the sample.

Variable	*N*	%
**Gender**		
Men	9	56.2
Women	7	43.8
**Education**		
X < 12	1	6.2
High school	8	50
Higher education (BA, MA, PhD)	7	43.8
**Diagnosis**		
Schizophrenia	4	25
Schizoaffective	5	31.2
Bipolar disorder	6	37.5
Major depressive disorder	1	6.3
**Duration of illness (DOI)**		
DOI < 2	1	6.3
2 < DOI < 5	1	6.3
5 < DOI < 10	4	25
DOI > 10	10	62.3
**Number of hospitalizations**		
X < 2	7	43.7
2 < X < 5	7	43.7
X > 10	2	12.5

*N* = 16.

### Procedure

The study was approved by Bar-Ilan University’s Committee for Ethical Research with Humans (#2021/01). Recruitment of participants was carried out over the course of a year at two community mental health centers and *via* website advertisements. Individuals who expressed an interest in participating in the study were first given an explanation about the study by a clinician who was working in the setting where they were receiving treatment, or by the research team if participants were responding to an advertisement. Those who agreed to participate were given detailed information about the study’s focus, procedure, and confidentiality issues, and all participants signed a consent form. All participants completed the MINI (34) for the DSM-IV, a short self-report form with some psychosocial and medical background information, and participated in an interview carried out by the first author (M.A). Interviews took place at the two mental health centers or in public places depending on the participants’ preferences, and lasted usually an hour. All interviews were recorded and transcribed, the content was kept confidential, and names or any identifying details were changed during the transcribing of the interviews to ensure anonymity. During the process of data collection and data analysis, the issue of adequate sample size needed for reaching saturation was constantly reviewed, based on the “information power” concept for qualitative research (35). In this process we took under consideration sample specificity, ensuring we had a representation of different diagnoses, of diverse patterns of medication use, and of sociodemographic background in our sample. Furthermore, another factor that contributed to the study’s information power was the quality of the dialog (35): The interviewer (first author) was a rehabilitation psychologist and PhD student with a background in working with people with SMIs, who was trained and experienced in conducting qualitative interviews.

### Instruments

1. The Mini-International Neuropsychiatric Interview/MINI (34) for the DSM-IV was used.
2. Sociodemographic and medical data were collected *via* self-report (see Table 1).
3. Pattern of medication use was assessed *via* self-report of the level of medication adherence on a 5-point scale, as in a previous study (36). This measure was carried out in order to ensure heterogeneity and representation of use patterns.
4. Semi-structured interview: The interview that was conducted derived from the narrative approach, and focuses on the story as the person chooses to tell it (37). Narrative approaches have been found to be especially relevant when assessing experiences of people in mental health recovery (38). First, participants were invited to share their experiences with psychiatric medications, from the moment the medications were first prescribed, and the process they underwent with these medications until the present time. In this part of the interview we aimed to capture participants’ unique experiences, thoughts, and feelings, and possible challenges in their process of coping with medication decisions. The second part consisted of specific questions which focused on possible factors that shape people’s attitudes and use patterns. Sample questions included: “What are your current beliefs about psychiatric medication?” or “What do you think about deciding to take or not to take medication?”

### Data analysis

**Figure 1:**
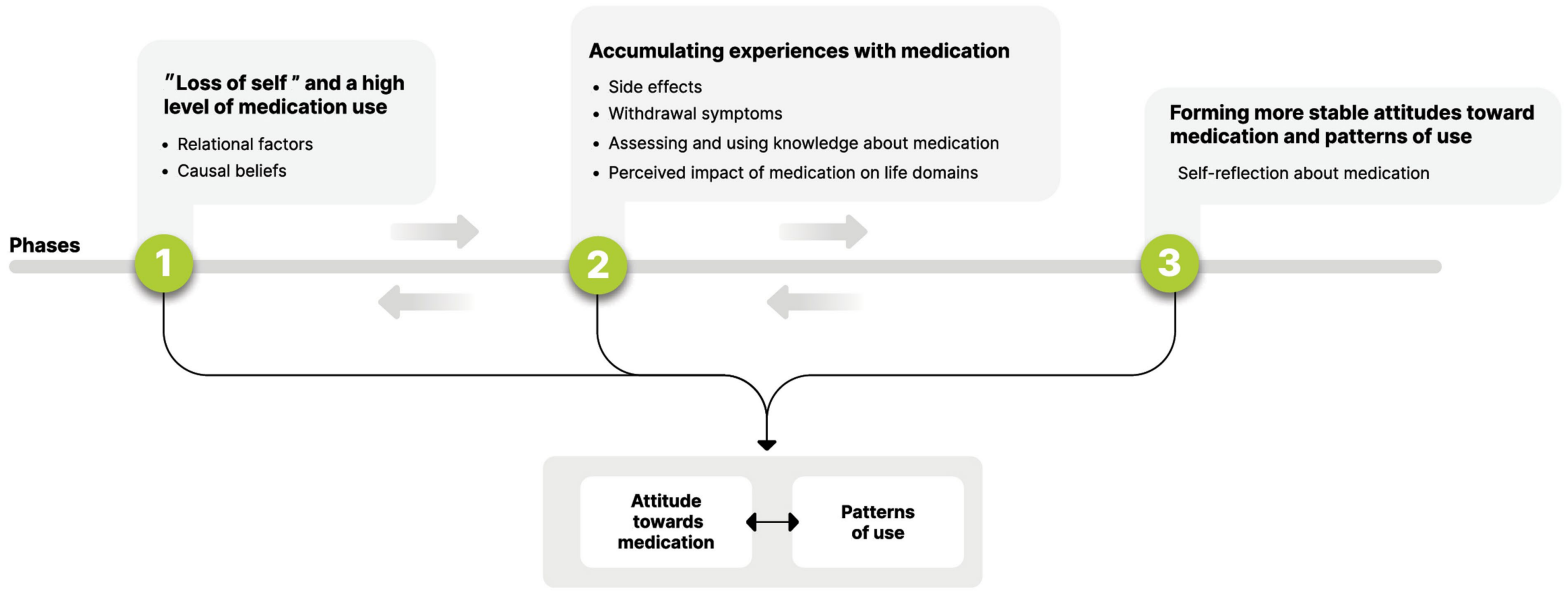
Longitudinal model of factors influencing attitudes toward medication and patterns of use.

Qualitative data were subject to thematic analysis using a narrative methodology, from a realistic point of view (i.e., reporting the meaning, experiences, and reality of the participants; 39). Data were analyzed in Hebrew and then translated into English. Data analysis followed the process of conceptualization including: (1) Reading the interviews several times to become familiar with the data. Data from all interviews were included in the analysis; (2) Interview data were coded for content line-by-line using the ATLAS.ti platform, with the marking of meaningful statements and quotations. This coding process was carried out by two judges (Authors 1 and 2), with Author 2 being masked to any information about the study’s participants or to the coding system developed. Furthermore, the order of the coding process between the interviews was different for each judge (i.e., Author 1 analyzed *via* the origin sequence, and Author 2 *via* a randomized order; 40). It can therefore be assumed that the coding and marking of meaningful units represented participants’ experiences rather than possible biases of the judges during the coding process; (3) Intercoder agreement analysis was performed, and reliability reached 88.8%. Discrepancies in coding were resolved through discussion until a consensus was reached; (4) All initial codes were classified under categories, for each interview separately. Categories that were the most prevalent and had the richest data were marked and labeled; (5) Categories from all interviews were then organized under main themes and subthemes. Of note, data collection and analysis were also performed simultaneously, until code saturation and meaning saturation were reached and no additional meaningful topics emerged (41); and (6) Building a model from the emerging themes (see Figure 1), which represent the factors that influence attitudes toward and patterns of use at different phases in participants’ narratives.

## Results

The analysis of the interviews yielded three discrete phases in the process of coping with medication prescription and adherence. Each phase was characterized by different dominant themes reflecting attitudes and patterns of use. Although the phases were sequential, the movement between them was fluid and dynamic, meaning that themes from previous phases could also be influential at later phases, and that changes in people’s relationships, illness course, or new experiences with medication could tilt the balance between the interacting themes and change the phase that the person currently occupied. The three phases were conceptualized by participants as: (1) “loss of self” and a high level of medication use; (2) accumulating experiences of using/reducing/stopping medication; and (3) forming more stable attitudes toward medication and developing one’s own pattern of use.

1. First phase: “Loss of self” and a high level of medication use

This phase refers to the period that came immediately after being diagnosed. During this phase there was no inner dialog about the use of medication but rather simple adherence. During this phase participants described a strong feeling of being engulfed by their illness, and a loss of identity and sense of being. Aviram for example shared:

If you had spoken with me a year ago, you would have seen that I could barely say a word. I was completely disconnected and had already given up on myself. I felt that my whole identity and inner experience was coffee and cigarettes. I lost my identity, my memory, total emptiness… I took the medication, although I think that at first I objected to taking medication, but then I was disconnected from this for several years.

Two themes emerged, which represent the dominant factors that affected attitudes toward medication and patterns of use during this phase: (1.1) relational factors, and (1.2) causal beliefs about mental illness.

1.1. Relational factors

All 16 of the participants (16 out of 16^1^) indicated the strong influence that their relationships with their prescribing psychiatrist or close family members and friends had on their attitudes toward medication and patterns of use. The nature of the relationship (i.e., both positive and negative aspects) with the psychiatrist was marked as an important factor with a strong effect on participants’ attitudes toward and patterns of use during this phase. Some participants marked in their narratives the positive aspects of the therapeutic alliance, such as open communication, receiving information, and emotional support from the psychiatrist. Others mentioned negative aspects including experiences of being criticized and judged, a lack of clarity and information given about the medication and side effects, and feelings of unresolved disagreements regarding desired processes or goals for treatment. These feelings often evoked intense mistrust and caused participants to hide information from their psychiatrists about changes they had made in their patterns of use or led them to reduce their follow-up visits. Aaron for example said that due to a disagreement with his psychiatrist regarding the focus of his treatment, he stopped seeing him on a regular basis and started to make changes in his patterns of use without supervision:

I wanted to decrease my medication and he [the psychiatrist] said: “The medication is not the issue here, leave that to me, just take them, what does it matter?” He didn’t ask me about my well-being or about the quality of my family life; that wasn’t the focus for him. He would try convincing me every time [to use medication], so I began to skip appointments and to see him less and less.

Anna said she spent 5 years of her life, after first experiencing symptoms, feeling that she was unseen by the mental health system. This feeling changed during the next phase when she started seeing a new treating psychiatrist with whom she had a much better therapeutic alliance. She described her experience during the first phase as follows:

I felt unseen, that my treating psychiatrists were changing constantly, that I was not being treated properly. This feeling was present for years… There was no option not to take the medication, it wasn’t a question at all! They [the psychiatrists] didn’t talk about the meaning of taking medication, about the possible side effects, about other alternatives that might have had fewer side effects for me… So I didn’t question it or ask myself if I should take the medication, I didn’t engage in any critical thinking at the time.

In addition to mentioning the importance of the relationship with the psychiatrist, participants emphasized the influence of a person close to them – a family member, spouse, or friend – who suggested considering a change in medication use. For example, Sarah described how at first she wasn’t aware of the degree to which the medication was having side effects on her, and she did not think about searching for alternatives. She described how her significant other took an important role in indicating the necessity for a change in her medication:

I wasn’t myself back then. I would go to the grocery store and forget why I had gone there, I would get on a bus and forget to get off. It was like I wasn’t part of this world. My partner noticed this; it was only thanks to her that they [the psychiatrists] changed my medication. She told my psychiatrist [what was happening] and really insisted [on a change in her medication], because I wasn’t aware of anything that was going on at the time.

1.2. Causal beliefs about mental illness

Many participants (9 out of 16) presented different causal beliefs about the onset of their symptoms, including trauma, toxic environments, being a highly sensitive person, the use of drugs, or biogenetic causes. Participants’ causal beliefs affected their attitudes toward medication in a range of ways. Those who believed biogenetic factors caused their illnesses tended to develop a more positive attitude toward medication use. For example, Gabriel said: “*If it’s a case which is chronic, like in my case, then I need the medication. In my case there is a family background; my grandmother and great grandmother also had bipolar disorder*.”

On the other hand, when participants believed there was a psycho-social explanation for their illness, their attitudes toward medication use were more complex. For example, Sharon, who believed her illness was caused by trauma, said:

I led a normal life for 30 years, and then I underwent a really bad event that was too difficult for my sensitive mind to handle. It doesn’t mean that I had been screwed up since birth. The psychiatrists always ask: “Who’s screwed up in the family? Its genetics.” Why genetics? Why suggest that this is the only explanation? Because you don’t have others? It seems a little irresponsible to me.

Although causal beliefs influenced participants’ attitudes toward medication, such attitudes were not necessarily reflected in their patterns of use, and adherence remained high during this phase. In sum, participants conceptualized the first phase after the onset of their illness as a period of “loss of self” characterized by strict medication adherence. During this phase they felt disconnected from themselves and from their identities. They shared the feeling that they did not trust their own decisions in general, and in regard to their care and medication specifically, and thus they tended to rely on their psychiatrist or others close to them (such as family and friends) who usually supported adherence during this phase. In addition, their causal beliefs about mental illness – which were also often influenced by their treatment team, family, and friends – was dominant during this phase and shaped their attitudes toward and actual use of medication.

2. Second phase: Accumulating experiences of using/reducing/stopping medication

After the initially high level of medication adherence post-mental-illness-onset, participants described attempts to reduce and/or stop medication use. Themes that emerged during the second phase, and which will be elaborated upon, included: (2.1) experiencing side effects, (2.2) experiencing withdrawal symptoms, (2.3) accessing and using knowledge about mental illness and medication, and (2.4) assessing and weighing the perceived impact of medication on various life domains, including functioning and symptoms, over time.

2.1. Experiencing side effects: was a theme mentioned by most participants (12 out of 16) as a factor that shaped a negative attitude toward medication. Participants mentioned many physical side effects, such as psychomotor retardation or restlessness, weight gain, and extreme tiredness. For example, John shared: “*I had been an athlete previously, and then I gained 30 kilos… I will never forget the moment that I met up with a childhood friend who barely recognized me… It was a wakeup call*.”

Moreover, participants emphasized various emotional side effects, such as the “zombie effect,” a feeling that they were “not themselves” because the medication restricted their range of emotion. Lili said:

The medication doesn’t allow you to have your full range of emotional experiences. It keeps you in a more restricted range. It's not like I haven’t experienced happiness or sadness with medication, but I feel like it limits my ability to connect to some internal parts of me that I love.

2.2. Experiencing withdrawal symptoms: was a theme noted by nearly half (6 out of 16) of the participants, who said they therefore felt they had become dependent on and/or addicted to the medication. This feeling, concordantly, had a negative influence on their attitude toward medication. Eve, for example, said: *“Nobody tells you how difficult the withdrawal symptoms are… For me the withdrawal symptoms started immediately, it began with dizziness and electric-shock sensations. It’s like a junky who needs the drug, the body goes through detox*.”

2.3. Accessing and using knowledge about mental illness and medication: was a theme mentioned by most of the participants (10 out of 16) as empowering them to form their own attitudes toward medication use. Gathering such knowledge was often an active process and shaped complex attitudes about medication stemming from different sources (books, research, exposure to other people’s experiences and first-person accounts), and not relying solely on the opinions that were so influential during the first phase, such as those of their psychiatrist, family, or friends. Participants emphasized how this process allowed them to feel a greater sense of self-efficacy, asserting their agency and arriving at a better understanding of and control over their treatment preferences and choices. For example, Tal shared her personal process of gaining knowledge and feeling empowered by it:

In the beginning I accepted that the illness was an illness for life, and the medication a medication for life… Then I read a lot of articles, did my own research, went to lectures. I think that for many years I gave a lot of authority to what my psychiatrists said, what was written on a piece of paper, and less authority to my personal strengths. Today this balance has changed.

2.4. Perceived impact of medication on life domains

Over time and with the accumulation of subjective experiences and knowledge about medication, conceptions about its impact on different life domains were formed. The growing appreciation of the impact of using or not using medication on key life domains such as family life, work, and severity of symptoms was also a factor that shaped participants’ attitudes toward and patterns of medication use.

Family functioning: Most participants (10 out of 16) described the impact of using, and more often stopping the use of medications, on their own behavior, which in turn influenced their interactions and relationships with family members. Gila shared how after the birth of her daughter she stopped taking medication, which led to a relapse for which she was hospitalized. She described how she currently struggles with her desire to stop taking medication and her family members’ response to that desire, based on the previous negative experience:

I think I'm getting closer to reducing my medication, but I'm also being careful about it, not reckless this time. I know it will scare my husband… I think my taking of medication gives him some peace and quiet right now because he is still traumatized from what happened after the birth. When I started to talk again about reducing the meds, my mom panicked at first… So I think that for now it's better to take them [the medication], just to be safe.

Work: Most of the participants (10 out of 16) displayed a range of opinions about the impact of taking medication vs. not taking medication on their work; some viewed it as a protective factor while others felt the opposite. Gabriel shared:

The thing about using medication for a long time is that you don’t always see the benefits of taking it. People are affected by the reward system, and you don’t see the reward when you’re in remission. Usually, people see the benefits of using medication only after experiencing several hospitalizations, like I did. I believe that waking up in the morning, and being able to function at work, not having to be hospitalized, that’s the true reward of using medication.

On the contrary, John felt that taking meds harmed his work performance:

My life stopped making any forward movement once I started with the meds and I began to be dependent on the status quo: the disability benefits, the social security, living with my parents… I would go to work and fall asleep during my shift. A lot of people were like that due to the medication.

Symptoms: Most of the participants (11 out of 16) reported developing their own opinion regarding the impact of medication use on their symptom severity. Some participants thought the medications were helpful, which provided a compelling reason to take the medications as prescribed. Daniel said:

The medications are necessary for me in order to maintain an ordinary life; they help me to survive. I don’t want to stop because every psychotic episode I had was worse than the one before. Today I have the confidence it won’t happen again, thanks to the medication I take.

At the same time participants such as Mona (in the following quote) also presented many questions and uncertainties about the relation between medication use and their symptoms:

I have tried so many medications, and things still don’t seem to be in balance. It’s really hard to tell if the medication is helpful or not. Did I just wake up moody? Is it the illness or is it the medication? The psychiatrist always asks me if it helped when we upped the dosage. Was it helpful? I have no idea what really helps and what affects what.

In sum, during the second phase and with the accumulation of experiences with medication, several factors emerged that had a powerful influence on attitudes toward medication and patterns of use. The constant interaction between the subjective experience of side effects and/or withdrawal symptoms, becoming knowledgeable about mental illness and medication, and the perceived impact of medication on different life domains over time generated many fluctuations in attitudes and patterns of use during this phase.

3. Third phase: Forming more stable attitudes toward medication and developing one’s own pattern of use

The third phase was characterized by coming to conclusions about the lived experience from the previous phases, resulting in more stable attitudes toward medication and actively choosing one’s own personal patterns of use.

Self-reflection about medication: Was a theme mentioned by the vast majority of participants (15 out of 16). Participants emphasized how important and valuable it was to reflect upon their overall experience with medication, evaluate trade-offs, and move toward a resolution that reflected a more stable attitude and pattern of use. When reflecting upon the experiences described in the previous phases, participants appraised what mattered most to them, weighed the pro and cons of using medication, and reflected on personal dilemmas or decisional conflicts that arose. This process generated two main approaches that reflect the resolution of a formed attitude and choosing a pattern of use: medication as a “crutch” and medication as a “cast.”

Medication as a “crutch” represents a perception of the long-term use of medication as a protective factor that enhances recovery. This view was found in the current study to be related to a positive attitude toward medication and higher adherence levels. Anna, for example, formed this perception about medication after much trial and error in her patterns of use, due to experiencing side effects as well as several changes in her treating psychiatrists. She shared that currently she feels safer with the support of medication: *“Today I feel that it [the medication] protects me. I think this feeling is meaningful in my recovery process. I think that it keeps me from falling… It feels as if it’s like grabbing onto something other than myself*.”

Medication as a “cast” represents a perception of medication as a short-lived and temporary form of support when symptoms begin to intensify. Participants who subscribed to this idea felt that medication use should not be ongoing or long-term because medication harms the recovery process in the long run and in the bigger picture. Udi, who stopped taking medication after having a powerful feeling of “not being himself,” and being influenced by things he had read and heard about medication and other treatment options, shared:

A lot of times taking medication is like putting on a cast. If your arm is broken, you put on a cast to fix it. Then the body goes through its own internal processes, which allows the bones to mend, and then you remove the cast. So medication, too, can also really help in different situations. But if someone’s arm is broken, and he continues to wear this cast for the rest of his life, what will happen? His arm will weaken, its strength and muscles will degenerate. In my view, that’s analogous to the long-term use of medication.

As mentioned earlier, the movement and transitions between different phases is dynamic, suggesting that the resolution during the third phase is not static or permanent, but rather can be influenced by new experiences, changes in symptom severity, and personal dilemmas that arise.

The movement between phases can be seen in the case of Aaron, a 41-year-old man who was diagnosed with bipolar disorder at the age of 23 and stopped taking medication 3 years prior to this interview. Throughout Aaron’s narrative, there is a constant shift between the significant themes that affected his attitudes and patterns of use, which depended on his own desires and the challenges he faced during different phases in his life. He described the first phase after illness onset as chaotic, having a psychotic episode followed by a prolonged depression during which he took his medications regularly. He mentioned that his psychiatrist’s opinion had a strong effect on him in terms of convincing him to take the medications:

The psychiatrist convinced me to continue taking my medication… But I always had some questions and doubts about how useful it was. Was the medication helpful? How could I tell what its impact on me was? I knew that I had been psychotic and then I wasn’t anymore. So, I did think the anti-psychotics were helpful. If I stopped taking my medications, would I become psychotic again? And I didn’t feel the anti-depressants were helping me because back then was when I had the most severe depressive episode I’d ever had.

During the second phase, Aaron had many fluctuations in his patterns of use including attempts to reduce, stop, and use again. This process was generated by the way he perceived the benefits and costs of using or not using medication for various life domains and what was most important to him at a specific time. For example, he stressed how the medication side effects, but mainly his engagement in peer support groups and exposure to critical attitudes toward medication, generated his own negative attitude toward medication and an attempt to stop use. On the other hand, he emphasized how important it was for him to be a reliable husband and parent:

When my attempts to stop using medication were irresponsible, my wife was against it. When I was high or down [emotionally], it really harmed her and my family. She was against the idea of stopping the meds when I was high and helped me to regulate use: not to stop use when I was already on the edge.

During the third phase, after he had accumulated a lot of experience with medication usage, his view of “medication as a cast” was formed. He concluded that the continuous use of medication was bad for him, and he started the process of discontinuing, but this time in a gradual manner and with support. In response to the interviewer’s question about what had shaped his current attitude, he shared his reflection about medication use as a complex trade-off and having to deal with the conflicts it generates:

On the one hand, there are the side effects, which make you want to stop or lower the medication dosage. I couldn't remember when I had last cried; you miss your emotions. But there is also a lot of fear, on both sides. I will compare it to my wife’s giving birth; she wanted to have a natural birth, mostly because she’s scared of hospitals. So what is more scary for her? The risk of a home birth or the fear of giving birth in a hospital? There is so much uncertainty, and this uncertainty might all be based on stories. For example, there were all kinds of stories about my depression, and how I behaved when I took the medication. Because I had episodes of mania and of depression when I took the medication, there were times I cried and others when I didn’t. It might also be that stories I internalized from an anti-psychiatric stance influenced me… It's too complex to make generalizations. The truth is more complicated than all of the stories.

Throughout Aaron’s narrative, it is clear how his subjective experiences generated his “medication as a cast” attitude and his attempts to withdraw from medication. Although Aaron eventually arrived at a more stable attitude, his narrative indicated several transitions between the second and third phase in both directions. This non-linear movement between phases occurred after new experiences with medication, the course of his illness (which included relapses), and his family life. All of these were significant themes and experiences that shaped his ongoing process of forming attitudes toward medication and developing his own pattern of use.

## Discussion

The present study reveals the complex and dynamic process of forming attitudes toward medication and decisional aspects regarding personal patterns of use. Our findings shed light on the process that people with an SMI undergo when psychiatric medication is prescribed for them and they must contend with dilemmas around adherence: from being overwhelmed by the onset of the illness and relying heavily on the treating staff and significant others (leading to high adherence) to taking a more active role in decision-making based on the accumulation of experiences and self-reflection (leading to personal preferences and a more stable attitude toward medication usage). This process progresses through distinct phases as proposed in our model (see Figure 1) and corresponds with Deegan and Drake’s (24) statement that compliance or adherence constructs fail to capture the complexity of decisions related to medication use, being active processes that entail many conflicts.

Our model in some ways resembles the Self-Efficacy Model of Medication Adherence (SEMMA), a theory-driven model which views adherence as a continuum, self-efficacy as a core influencing element, and the interaction between different variables (e.g., perceived medication efficacy, personal issues, social stigma) as exerting an overall influence on people’s adherence level (33). First, similar factors were found to be influential on patterns of use in both models, such as perceived medication efficacy, relationships with health professionals or significant others, and medication side effects (33). Second, both models refer to adherence as a continuum, what we have termed “patterns of use,” reflecting the heterogeneity embedded in the construct of adherence. Nonetheless, two fundamental differences should be noted. Our model expands on SEMMA’s placing the person at the heart of the approach by referring to people’s attitudes toward medication as a core element in the model in addition to people’s actual patterns of use. This distinction is important as it reveals possible complex conflicts within people, when different factors influencing their attitudes and patterns of use tilt the balance and might generate a pattern of use which is quite opposite to people’s stated attitude toward medication. This conflict was illustrated in the case study presented, for example when Aaron had a negative attitude toward medication but was adherent because of his wife’s opinion and his understanding of how withdrawal could have negative effects on his family. Referring to patterns of use alone would discount the complexity embedded in decisions about medication use, which are highly influenced by people’s attitudes (7). The third phase of our model (see Figure 1), which reflects people’s thoughts about medication and evaluating the trade-offs of use vs. non-use, represents this complexity within people’s decision-making processes. Extending the focus from factors influencing adherence to understanding people’s attitudes, beliefs, and values regarding medication use can also help to promote shared decision-making, a process wherein clinicians and patients work together, recognizing and respecting patients’ preferences and role in managing their health (42).

The second way in which our model differs from current and classic models for understanding adherence among individuals with an SMI (e.g., 30, 32, 33, 43), and with utmost significance, is that it emphasizes longitudinal aspects and dynamic processes: namely, in each phase different themes interact with each other and exert a greater influence on people’s decision-making processes. The model indicates that there is no single element that is more influential than another in its total effect on attitudes and usage patterns; rather, one element may be more dominant than another at a given timepoint, depending on the individual’s personal evaluation of its significance. Although the model emphasizes distinctive sequential phases, it also represents a non-linear process where the movement between phases is dynamic, and different experiences (i.e., relapses, changes in the family status, occupation, gaining new knowledge about medication) can create movement back to previous stages.

**Figure 2:**
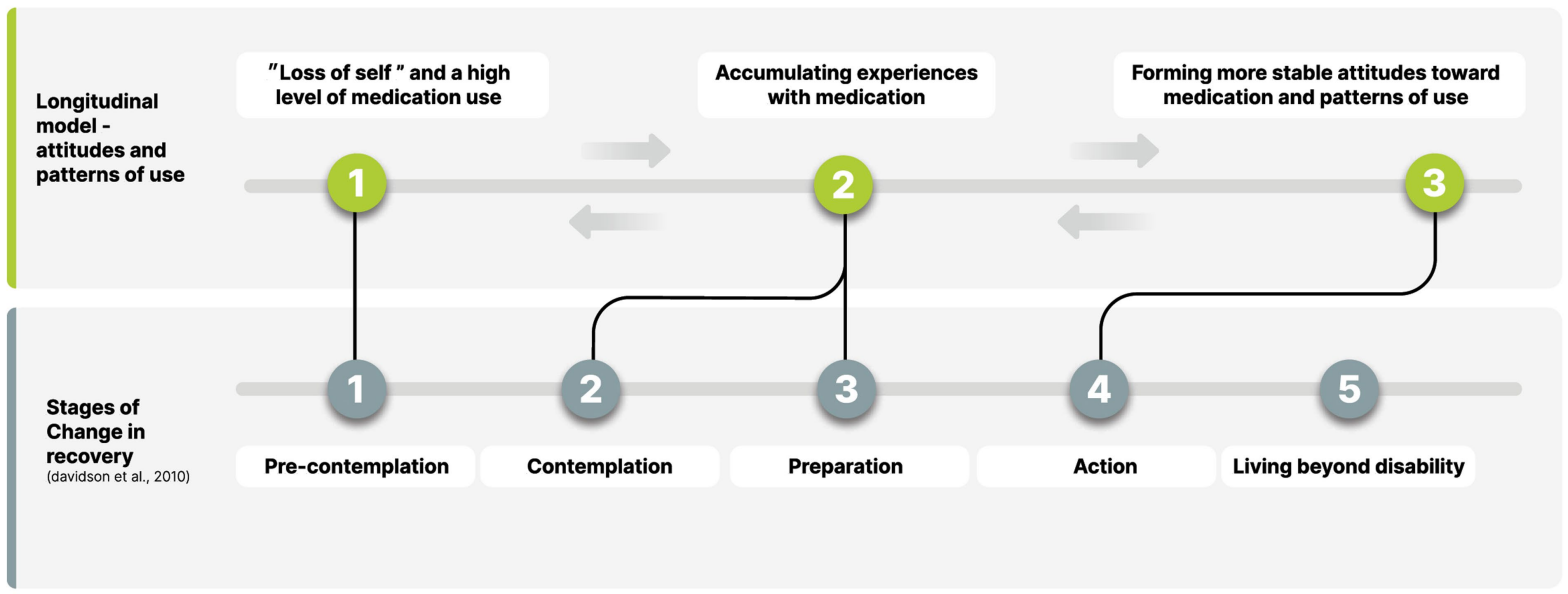
Comparison to stages of change in recovery model.

Reframing medication decision-making as a dynamic, non-linear process is a potentially important conceptual development with several implications. First, such reframing is in line with other prominent and influential frameworks that have emphasized mental health recovery as a non-linear dynamic process (19, 44–46). Second, this model aligns with other models that indicate discrete phases in recovery processes (e.g., 47–49). Davidson and colleagues (47) offered a model that outlined stages of change in mental health recovery but emphasized the limited nature of this model, as it excludes the non-linear nature of recovery. They identified five distinct stages, a few of which correspond with the current model of forming attitudes toward medication and patterns of use (see Figure 2). The first stage, termed “pre-contemplation” or “pre-recovery,” is characterized by feelings of powerlessness, confusion, despair, and a feeling that the self is overwhelmed by and immersed in the disorder. This stage mirrors the first stage in the present model: the “loss of self” and high levels of medication use. Identifying this distinctive phase in the process of forming attitudes toward and patterns of medication use is critical, as it emphasizes the time period after illness onset, when the patient might be overwhelmed with new experiences, and decisional processes seem to require the involvement of others (formal or informal). This notion was expressed by participants in the current study, sharing their limited ability to make decisions during this phase and their need for assistance from psychiatrists, family, and friends.

The next two phases in the “stages” model of change in recovery (47) – “contemplation,” which is the growing awareness of a desirable behavioral change, and “preparation,” which is planning for a change and a transformation of the self from passive to active – resemble the second stage of our model: “accumulating experience of using/reducing/stopping medication.” This transition from passive to active can be seen for example in the transformation from causal beliefs, a relatively passive process of attribution for the illness cause (50) during the first stage, to an active search for knowledge and understanding, as seen in the second phase. This transformation to having an active role was marked by participants as empowering in their process of forming attitudes toward medication and choosing one’s own pattern of use.

Last, the “action” phase in recovery processes (47) reflects an intentional effort to cope with the illness and work on personal goals. This phase is reflected in our final phase, when the person forms an integrated and stable attitude toward medication use, representing their personal preferences about treatment and values and putting them into action when choosing their pattern of use. It is not surprising that there are parallel processes and many similarities between recovery stages, and the process of coping with medication prescription and adherence decisions, when forming personal attitudes toward medication. Being able to reflect upon treatment preferences and options, having choices, and taking control of one’s life are fundamental aspects of recovery in mental health.

### Limitations

The current study had several limitations. First, although the sample size was adequate, it was nevertheless relatively small; as such, generalizing from the results should be done cautiously. Furthermore, it is possible that due to the small sample size, significant themes that have been found in the literature as being influential on people’s patterns of use (e.g., perceived stigma; substance abuse; 3, 7) were not sufficiently represented in the current data set. Second, the study applied a retrospective design, in which participants were asked to share their experiences with medication from illness onset until the present; recall bias thus undoubtedly exerted an influence, especially as many of the study participants had long duration of illness. Third, as recruitment was carried out in different settings simultaneously, and during the COVID-19 pandemic, the interviews took place in different settings (see Procedure) which might have influenced participants’ ability to openly share difficult issues in their personal narratives.

### Implications for theory, research, and practice

The proposed longitudinal model of factors influencing attitudes toward medication and patterns of use emphasizes the different challenges in the complex process of forming such attitudes and choosing patterns of use. The model comprises three distinctive phases of attitude formation and consequent pattern of use over the course of many years and decades of coping with an SMI. Several implications of the model should be considered. First, the growing understanding that adherence is not a dichotomous construct but rather a continuum (33) with different patterns of use is crucial for theory and practice. There is a wide range of ways in which people choose to use their psychiatric medication, and these ways are subject to change over time. In addition, we must aim to change the perception of non-adherence as being necessarily a negative phenomenon; rather, it should be accepted as a treatment preference and a legitimate life choice (51). Second, clinicians should be encouraged to try to identify the phase their patients currently occupy in the process of forming attitudes toward medication and choosing patterns of use. Identifying the phase will enable clinicians to better understand possible challenges and related themes that affect their patients’ attitudes and patterns of use and establish a joint reflective dialog to enhance alliance and shared decision-making. Finally, broader, more flexible, and nonlinear models of the process of forming attitudes toward medication and choosing patterns of use are needed to deepen our understanding of the changes and difficult issues that arise when coping with medication prescription and adherence decisions.

Going forward, researchers should aim to develop tools that refer to and measure patterns of use and attitudes toward medications as complex concepts. We should also adapt qualitative measures for measuring adherence and take into consideration the personal significance of different factors that influence people’s attitudes toward medication. Models should refine and try to accurately assess the complex interaction between factors influencing attitudes toward medication and patterns of use, with a longitudinal design and with heterogeneity in participants’ duration of illness, as different phases in coping with an SMI and medication use raise different challenges and dilemmas over time.

## Data availability statement

The raw data supporting the conclusions of this article will be made available by the authors, without undue reservation.

## Ethics statement

The studies involving human participants were reviewed and approved by the Bar-Ilan University’s Committee for Ethical Research with Humans (#2021/01). The patients/participants provided their written informed consent to participate in this study.

## Author contributions

MA interviewed all the participants and wrote the first draft of the manuscript. MA and DR analyzed the qualitative data. DR and IH-O contributed in editing and revising the manuscript. All authors took part in conceptualizing the ideas and planning the research procedure.

## Funding

This study was funded by the Israel Science Foundation (ISF; 2545/21).

## Conflict of interest

The authors declare that the research was conducted in the absence of any commercial or financial relationships that could be construed as a potential conflict of interest.

## Publisher’s note

All claims expressed in this article are solely those of the authors and do not necessarily represent those of their affiliated organizations, or those of the publisher, the editors and the reviewers. Any product that may be evaluated in this article, or claim that may be made by its manufacturer, is not guaranteed or endorsed by the publisher.

## Footnotes

^1^All numbers in themes and subthemes refer to the number of participants who mentioned the related subject in their narrative.
